# The acute and repeated bout effects of multi-joint eccentric exercise on physical function and balance in older adults

**DOI:** 10.1007/s00421-023-05226-z

**Published:** 2023-05-22

**Authors:** Brett A. Baxter, Anthony W. Baross, Declan J. Ryan, Ben H. Wright, Anthony D. Kay

**Affiliations:** Centre for Physical Activity and Life Sciences, Faculty of Art, Science and Technology, University of Northampton, Northampton, NN1 5PH UK

**Keywords:** Eccentric contractions, Strength, Delayed onset muscle soreness, Ageing

## Abstract

**Purpose:**

Eccentric muscle actions generate high levels of force at a low metabolic cost, making them a suitable training modality to combat age-related neuromuscular decline. The temporary muscle soreness associated with high intensity eccentric contractions may explain their limited use in clinical exercise prescription, however any discomfort is often alleviated after the initial bout (repeated bout effect). Therefore, the aims of the present study were to examine the acute and repeated bout effects of eccentric contractions on neuromuscular factors associated with the risk of falling in older adults.

**Methods:**

Balance, functional ability [timed up-and-go and sit-to-stand], and lower-limb maximal and explosive strength were measured in 13 participants (67.6 ± 4.9 year) pre- and post-eccentric exercise (0, 24, 48, and 72 hr) in Bout 1 and 14 days later in Bout 2. The eccentric exercise intervention was performed on an isokinetic unilateral stepper ergometer at 50% of maximal eccentric strength at 18 step‧min^−1^ per limb for 7 min (126 steps per limb). Two-way repeated measures ANOVAs were conducted to identify any significant effects (*P* ≤ 0.05).

**Results:**

Eccentric strength significantly decreased (− 13%) in Bout 1 at 24 hr post-exercise; no significant reduction was observed at any other time-point after Bout 1. No significant reductions occurred in static balance or functional ability at any time-point in either bout.

**Conclusion:**

Submaximal multi-joint eccentric exercise results in minimal disruption to neuromuscular function associated with falls in older adults after the initial bout.

## Introduction

Resistance training in older populations improves muscle size, strength, explosive capacity (rate of torque development [RTD]), and functional ability (LaStayo et al. 2003; Caserotti et al. 2008; Borde et al. 2015; Lopez et al. 2017; Grgic et al. 2020), which can decrease the risk of falling (Sherrington et al. 2017). The magnitude of adaptation will largely depend upon the training type, intensity, volume, programme duration, but also the capacity of the individuals to perform and tolerate the exercise (Steib et al. 2010; Fragala et al. 2019). Eccentric resistance training has been increasingly researched over the past 20 years due to its high-force low-metabolic cost characteristics, making it suitable for a range of exercise intolerant clinical populations with neuromuscular and cardiovascular comorbidities (LaStayo et al. 2014; Hoppeler 2016; Douglas et al. 2017; Hody et al. 2019). Furthermore, the superior muscular adaptations induced following eccentric training compared to other contraction modes (Roig et al. 2009; Schoenfeld et al. 2017; Douglas et al. 2017; Chen et al. 2017; Kulkarni et al. 2021) make it ideal for clinical exercise prescription for older adults to prevent or reverse age-related neuromuscular decline and to preserve functional ability.

Notwithstanding the beneficial chronic adaptations from eccentric exercise, detrimental short-term effects often termed exercise-induced muscle damage (EIMD) can occur in the days after eccentric exercise (Hornberger and Chien 2006; Coffey and Hawley 2007; Schoenfeld 2010). Symptoms of EIMD include delayed onset muscle soreness (DOMS), elevated blood serum molecular markers (e.g. creatine kinase), muscle weakness, and impaired balance (Hyldahl and Hubal 2014; Hody et al. 2019; Hill et al. 2020). Reductions in peak strength have been labelled as the most reliable measure of EIMD (Hyldahl and Hubal 2014) as they represent damage to the neuromuscular system. However, it takes ~ 300 ms to reach peak strength (Aagaard et al. 2002; Aagaard 2003) but only 70–120 ms to fall (Lockhart 2013). Therefore, whilst peak strength is a reliable EIMD measure, functional performance (timed up-and-go [TUG] and sit-to-stand [STS]), balance, and explosive ability (RTD and contractile impulse) are required to maintain autonomy (Hughes et al. 1996; Christensen et al. 2006; Jellesmark et al. 2012) and reduce the risk of falling (Aagaard et al. 2002; Korhonen et al. 2006; Bento et al. 2010; Quinlan et al. 2018), hence these may be more meaningful outcome variables to determine acute detrimental effects in older adults.

Following the initial exposure to eccentric exercise (even at submaximal intensities), EIMD is often diminished (Chen et al. 2012a, b), which is likely attributable to the repeated bout effect (RBE) phenomenon (Clarkson et al. 1992). Often, laboratory-based studies that intend to induce substantial EIMD use maximal eccentric contractions (Nosaka et al. 2001a; Chen et al. 2011), upper-limb muscles less exposed to high-intensity eccentric loading (Nosaka et al. 2002; Chapman et al. 2008; Chen et al. 2012b, 2014), or uni-joint exercises to increase the loading on a specific muscle group (Byrne et al. 2001; Hody et al. 2013; Tseng et al. 2016). The RBE may improve tolerance to eccentric exercise and thus, potentially increase adherence by alleviating or eliminating the negative symptoms associated with EIMD. However, these dosages, muscle groups, and intensities may not be suitable for older adults to reduce fall risk. Given the importance of exercise intensity to minimise symptoms of EIMD, and of exercise specificity to enable the transition of training adaptations to functional ability (Kraemer et al. 2002), it is crucial to understand the impact of submaximal lower-limb multi-joint exercises that can replicate activities of daily living.

To safely prescribe eccentric exercises to older adults, the acute temporal responses induced by multi-joint eccentric exercise must be fully understood, particularly those that could compromise activities of daily living and increase the risk of falling (Picorelli et al. 2014) such as strength, explosive strength balance, and the ability to perform functional tasks. Therefore, the aims of the present study were to examine the magnitude of EIMD elicited by a submaximal multi-joint eccentric exercise bout in older adults on measures of functional ability and explosive capacity. A secondary aim was to examine the magnitude of protection (RBE) following a second bout of identical submaximal eccentric exercise performed 14 days later. It was hypothesised that there would be a significant change in neuromuscular performance (static balance, functional ability, strength, and explosive capacity) and perceptual measures (perceived exertion and muscle soreness) after Bout 1 but not after Bout 2.

## Methods

### Participants

Thirteen community-dwelling independently-living older adults ([4 male, 9 female] age = 67.6 ± 4.9 year, height = 1.7 ± 0.1 m, mass = 71.2 ± 14.7 kg, body mass index = 25.5 ± 4.8 kg·m^−2^) volunteered for the study and were recruited via word of mouth, radio broadcast, and leaflet handouts. Participants were excluded if they had a lower-limb musculoskeletal injury within the past three months, were resistance trained, or were taking any anti-inflammatory medication during the time of participation. All participants provided written informed consent and completed a medical questionnaire before the commencement of data collection. Ethical approval was gained from the University Research Ethics Committee with all procedures conducted in accordance with the Declaration of Helsinki.

### Protocol overview

**Fig. 1:**
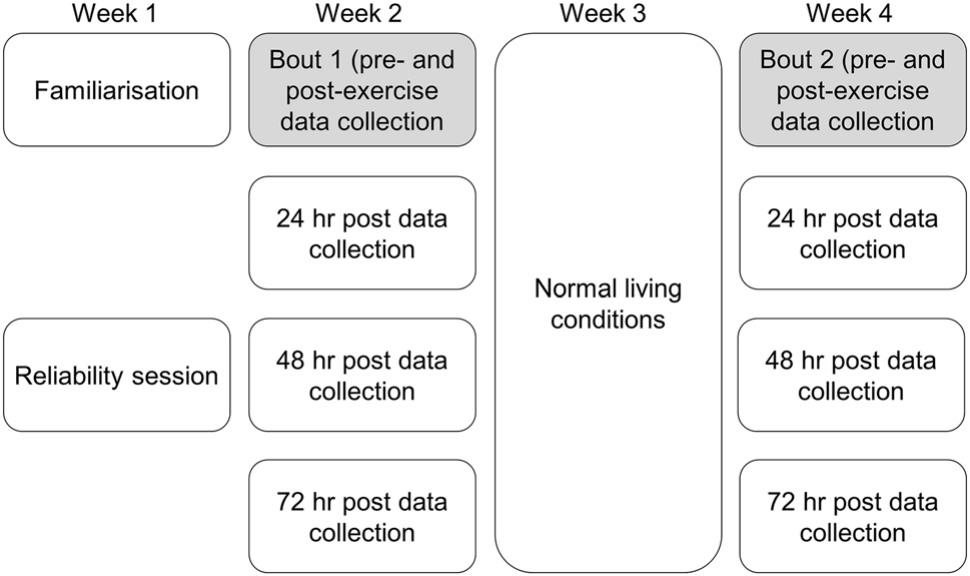
A schematic of the study design; where each box represents a day/data collection session and grey boxes represent sessions that included a bout of eccentric exercise. Participants were familiarised before undertaking two bouts of eccentric exercise, separated by 14 days, whereby data collection was undertaken pre-exercise, immediately post, 24, 48, and 72 hr post-exercise

Participants visited the laboratory on 10 occasions over four weeks (Fig. 1) with the first week including a familiarisation session followed 48 hr later by an initial data collection session. During the first visit, participants performed the eccentric force assessments (two sets of 12 repetitions) at 25% of their perceived maximal force, which increased to 50% in their second session to familiarise themselves with the protocol. Maximal eccentric force was excluded from the familiarisation session and initial data collection session to minimise potential RBE from familiarisation influencing the study (Nosaka et al. 2001b).

**Fig. 2:**
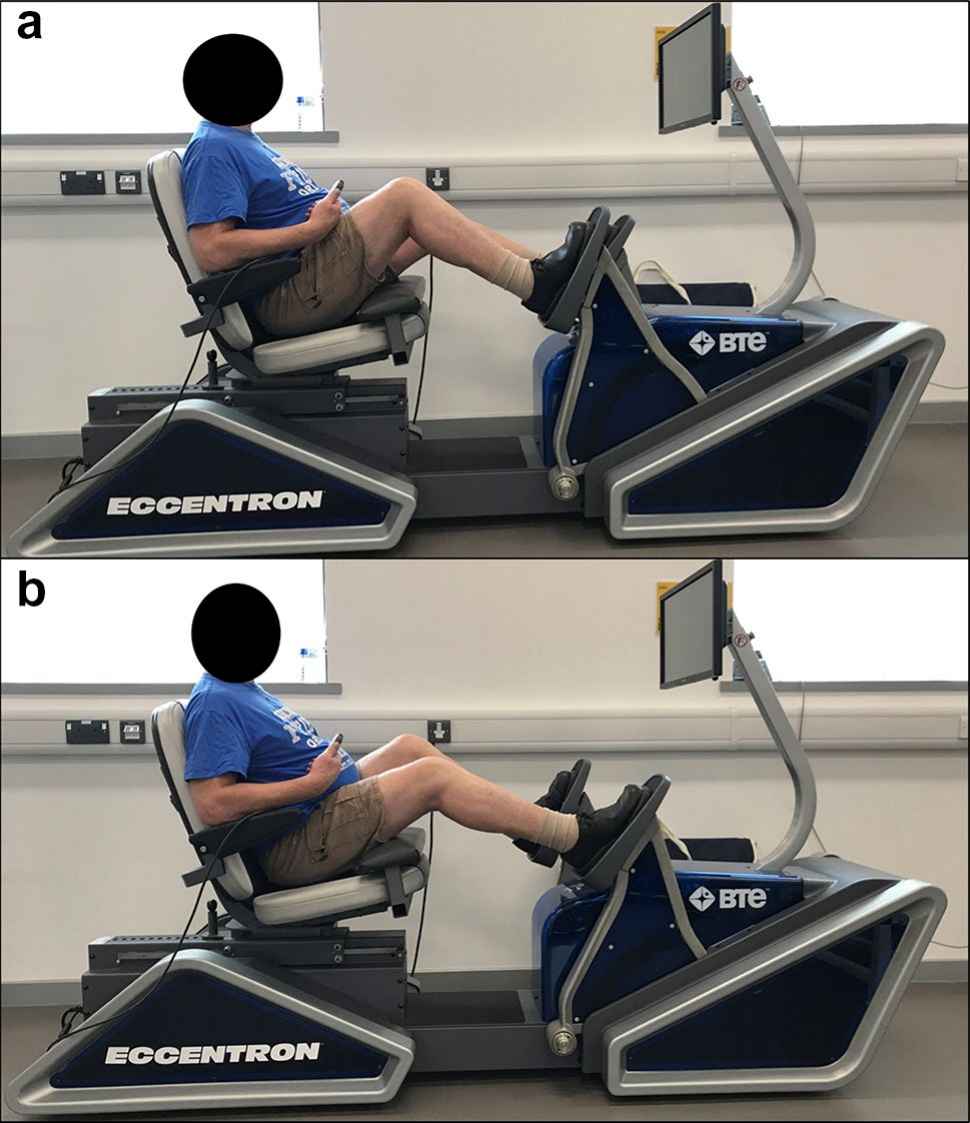
The isokinetic stepper ergometer used to perform eccentric exercise and assess maximal eccentric force. Panel **a** displays the most flexed position of the right knee whilst panel **b** displays the most extended position of the right knee

During week 2 (Bout 1), the data collection session was repeated to assess between-session reliability (2nd and 3rd visits) and provide pre-exercise baseline data (3rd visit) on neuromuscular function and perceptual measures (described later). Immediately after the data collection session (3rd visit), participants completed the eccentric exercise protocol (described later) on a recumbent isokinetic stepper ergometer (Eccentron, Baltimore Therapeutic Equipment, Hanover, MD, USA [Fig. 2]). No testing was conducted in week 3; in week 4 (Bout 2; 7th–10th visits) the data collection session and eccentric exercise protocol (identical to week 2) were repeated, providing 14 days between bouts of eccentric exercise to examine the RBE.

### Eccentric exercise intervention

Before the eccentric exercise protocol, the seat was adjusted so the knee could not extend more than 150° (180° = full extension) and the stride position was set so that the knee did not flex to less than 90° to minimise possible injury, with a stop button handed to the participant allowing the test to be terminated at any point. To perform the eccentric exercise the footplates on the stepper ergometer moved towards the participant in an alternating manner (i.e. as one footplate moved towards the participant the opposing footplate moved away). Participants were instructed to resist the footplate unilaterally, alternating between limbs as the footplate moved towards them, resulting in an eccentric contraction of the hip extensors, knee extensors, and plantar flexors and to relax as the footplate moved away, allowing the extensor musculature of one limb to contract at any given time throughout the exercise. The exercise was performed for 7 min at a constant rate of 18 step·min^−1^ per limb (126 repetitions per limb) at 50% maximal voluntary eccentric force for 5 min with a 1-min warm-up and cool down at 25% maximal voluntary eccentric force. During the eccentric exercise, real-time visual display was provided on-screen that allowed participants to stay in rhythm with the stepper and visualise the accuracy of force application with reference to a pre-set target level and acceptable range (40–60% maximal eccentric force). Metrics of neuromuscular function and perceptual measures (described below) were collected pre-exercise, immediately after (0 hr), and 24, 48, and 72 hr (4th–6th visits) post-exercise, with all metrics measured at the same time of day (± 1 hr).

### Measures

#### Muscle soreness

Palpation muscle soreness was assessed in a standing position via palpation of the proximal, mid-belly, and distal portions of the right vastus lateralis with the mean of the three sites used for subsequent analysis (Lavender and Nosaka 2008a). Movement soreness was assessed by asking the participant to perform a squat movement to approximately 90° of knee flexion. During palpation and the squat movement, participants rated their muscle soreness on an 11-point visual analogue scale (0 = “no pain”; 10 = “worst pain possible”). To eliminate the impact of testing procedures influencing measures of muscle soreness, these data were collected at the beginning of each session.

#### Balance

Postural sway (anteroposterior and mediolateral displacement [cm], 95% ellipse area [cm^2^], path length [cm], and centre of pressure velocity [cm·s^−1^]), alongside maximum anterior displacement [cm] were assessed on a force platform (AMTI, AccuGait, Watertown, MA, USA) with data sampled at 100 Hz and analysed using BioAnalysis v.2.2 software (AMTI). Participants were instructed to allow their arms to hang freely by their sides and look straight ahead at a target 1.5 m away, adjusted to the participant’s eye level. The researcher stood close by for each trial to provide support in the event of a loss of balance but maintained distance to prevent a distraction affecting natural sway. To assess postural sway, quiet stance trials were performed for 30 s, unshod with the feet shoulder-width apart. Participants’ postural sway was assessed during eyes open and eyes closed conditions, performed in a counterbalanced order (three trials for each condition) with the mean of the three trials in each condition used for subsequent analysis. The reliability and validity of these postural measurements have been established previously for this sampling epoch (Pinsault and Vuillerme 2009). Maximum anterior displacement was assessed by instructing participants to lean as far forwards as possible without using their arms for stabilisation, three trials were conducted with the longest value used for subsequent analysis.

#### Functional ability

TUG was used to assess functional mobility and required the participants to rise from a chair without the use of their arms, walk three metres, turn around, walk back, and sit on the chair. Participants were instructed to walk at a self-selected “comfortable” pace to prevent rushing and to replicate everyday behaviour. Participants completed three trials separated by a 1-min rest, the time to complete the test was recorded to the nearest 0.01 s with the fastest trial used for analysis. For the indirect assessment of lower-limb muscular power, participants completed the 10-repetition STS. The time to complete 10 repetitions was recorded to the nearest 0.01 s using a stopwatch (the trial ended when the participant was fully stood up on the 10^th^ repetition). The assessment was performed twice with a 1-min rest between trials with the fastest trial used for subsequent analysis.

#### Contractile ability

RTD (N·ms^−1^), contractile impulse (N‧m‧s), and maximal knee extensor torque (N‧m) were assessed using isometric dynamometry with the participants’ right lateral femoral condyle aligned with the axis rotation of the dynamometer (Biodex System 3 Pro, IPRS, Suffolk, UK), hips flexed to 95° (180° = full extension), and the knee flexed to 110°; i.e., the approximate angle whereby peak knee extensor strength is produced in older adults (Yoon et al. 1991; Frey-Law et al. 2012). Participants performed three submaximal unilateral isometric contractions at 50 and 75% of perceived maximum, with arms folded across the shoulders during all contractions and non-elastic strapping over the waist to minimise extraneous movement. Immediately prior to initiating the test, participants were instructed to develop a small level of pre-tension (4.55 ± 1.13 N·m) to reduce the amount of force dissipation into the cushioning on the lever arm (Tillin et al. 2013). Following submaximal efforts, participants performed five rapid contractions as “*fast* and hard” (with the emphasis on fast) as possible, with each contraction separated by 15 s rest. If a trial displayed signs of countermovement (visually checked for an initial reduction in torque), the trial was repeated. RTD and impulse data were extracted from the five explosive contractions with the mean of the three most explosive trials (greatest RTD) used for subsequent analysis (Maffiuletti et al. 2016).

RTD was calculated from the torque-time trace (Δtorque‧Δtime^−1^), alongside impulse (∫torque d*t*) over numerous epochs from the onset of contraction (0–100, 0–150, 0–200, 0–250, 0–300 ms); RTD between 100 and 200 ms (RTD_100-200_) was also examined as it may be more representative of EIMD (Peñailillo et al. 2015). Peak RTD (RTD_peak_) was examined using a rolling 20-ms epoch (Haff et al. 2015). The onset of muscular contraction (0 ms) was determined manually using visual inspection of the inflexion point on the torque-time trace in a figure with a y-axis (torque) scale of ~ 1 N·m and x-axis (time) scale of ~ 200 ms (Tillin et al. 2010).

Following the rapid contractions, participants then performed a ramped maximal voluntary isometric contraction initiated from rest with participants instructed to push “as hard as possible” over a 5-s epoch. Following a 1-min rest period, the participants repeated the contraction until three valid (no observable countermovement on the force trace) trials were collected. The highest value of isometric torque (N‧m) from the three maximal trials was used for subsequent analysis.

Joint torque data during these trials were directed from the dynamometer to a high-level transducer (HLT100C, Biopac, CA, USA) before analogue-to-digital sampling at 2000 Hz (MP150 Data Acquisition, Biopac, CA, USA). The data were then directed to a personal computer (Elitebook, HP Inc., CA, USA) running AcqKnowledge software (v.4.4, Biopac). Subsequently, data were smoothed in RStudio (v.1.0.153, RStudio, Inc., MA, USA) off-line with a custom-written fourth-order, zero-lag Butterworth filter at 150 Hz (Thompson 2019).

Eccentric lower-limb force (N) was assessed on a recumbent alternating unilateral isokinetic stepper ergometer (Eccentron). Participants performed two submaximal warm-up sets (two sets of 12 repetitions) at 25 and 50% of their maximal effort, followed by two sets of 12 maximal efforts, which were separated by a 1-min rest. The highest value of eccentric force was used for subsequent analysis.

#### Perceived exertion

Rating of perceived exertion (RPE) was recorded following the eccentric exercise session and for each trial of the functional tasks (STS and TUG) using the Borg CR10 scale (Borg 1998). The mean of the trials was calculated and reported as the RPE for each task.

#### Index of protection

To determine the “Index of Protection” elicited by the RBE, the magnitude of change (%) in variables from pre- to 24 hr post-exercise was calculated following Bout 1 and Bout 2. Twenty-four hr post-exercise was chosen because this may be a better representation of EIMD as it reduces the influence of fatigue (Chen et al. 2014). Once the magnitude of change had been calculated from pre- to 24 hr post-exercise for both bouts, the index of protection was calculated as:

$${\text{Index of}}\;{\text{Protection}}(\% ){\mkern 1mu} = {\mkern 1mu} \frac{{({\text{ECC}}1 - {\text{ECC}}2)}}{{{\text{ECC}}1}} \times 100$$

where ECC1 = the magnitude of change (%) between pre- and 24 hr post-exercise following the initial bout of exercise, and ECC2 = the magnitude of change (%) between pre- and 24 hr post-exercise following the second bout of exercise; e.g. if strength decreased 24 hr post-exercise by 30% after Bout 1 and by 15% after Bout 2, the index of protection would be 50%.

### Statistical analyses

All data analyses were conducted using SPSS for Windows (v.28 IBM Corp., NY, USA) with group data reported as mean ± SE and change data reported as mean ± SD. A participant missed one testing session (Bout 1, 72 hr post-exercise) so multiple imputation was conducted (Kang 2013) using the automatic method on SPSS to prevent the participant being removed from the analyses via listwise deletion. Five imputations were conducted following the calculation of the fraction of missing information (Bodner 2008). Normal distribution was examined using Shapiro–Wilk tests with transformation (logarithm base 10) performed where data failed the assumption of normal distribution. Data that continued to violate normal distribution were analysed using non-parametric Friedman and Kruskal–Wallis tests to examine within- and between-variance, respectively, alongside Wilcoxon tests to compare within-subject non-parametric differences. Normally distributed data were analysed using a two-way repeated measures ANOVA to examine the within-subject effects of time (× 5 [Pre-**,** 0, 24, 48, and 72 hr post-exercise]) and Bout (× 2 [Bout 1 and Bout 2]). Homogeneity of variance was assessed using Mauchly’s test of sphericity with a Greenhouse–Geisser correction used if sphericity was violated. Post-hoc analyses of non-parametric data were conducted via traditional pairwise comparisons and post-hoc pairwise comparison analyses of parametric data were conducted using Tukey’s Honestly Significant Difference Test within each respective bout. For parametric data, η_p_^2^ was used to assess the magnitude of the effect for the two-way repeated measures ANOVA. For pairwise comparisons, standardised differences were calculated to examine the magnitude of change for all significant findings; for non-parametric data *r* was calculated (Field 2009) and for parametric data change-score repeated measures Cohen’s *d* (*d*) was calculated (Morris and DeShon 2002). Statistical significance for all tests was accepted at *P*   0.05.

### Reliability

#### Within-session reliability

To determine within-session reliability, coefficients of variation (CV) and intraclass correlation coefficients (ICC_3,1_) were calculated for all variables on trials within the pre-exercise session during Bout 1 (visit 3). Following the guidelines of Koo and Li (2016), an ICC < 0.50 was considered poor, 0.50–0.74 moderate, 0.75–0.90 good, and > 0.90 excellent. Metrics of balance displayed moderate-to-excellent reliability for measures of anteroposterior and mediolateral displacement, 95% ellipse area, path length, centre of pressure velocity, and maximum anterior displacement (ICC = 0.74–0.98; CV = 6.4–12.6%). STS and TUG time displayed moderate-to-good reliability (ICC = 0.79–0.88; CV = 5.9–9.5%). Dynamometry displayed excellent reliability for maximal isometric torque (ICC = 0.96; CV = 5.7%) and good reliability for maximal eccentric force (ICC = 0.88; CV = 11.9%). RTD and impulse ≥ 100 ms displayed good-to-excellent reliability (ICC = 0.90–0.96; CV = 6.3–14.9%), however RTD and impulse ≤ 50 ms displayed poor-to good reliability (ICC = 0.47–0.71) with large CV’s (20.3–30.9%).

#### Between-session reliability

To calculate between-session reliability, metrics of balance, TUG and STS time, maximal isometric torque, RTD, and impulse data were taken during the second and third visit. To minimise potential RBE effects influencing the data, maximal eccentric force was excluded from the between-session reliability analyses. Metrics of balance displayed excellent reliability for measures of anteroposterior and mediolateral displacement, 95% ellipse area, path length, centre of pressure velocity, and maximum anterior displacement (ICC = 0.91–1.00; CV = 2.6–10.1%). STS and TUG time displayed excellent reliability (ICC = 0.98; CV = 2.6–3.9%). Dynamometry displayed excellent reliability for maximal isometric torque (ICC = 0.98; CV = 2.5%). RTD and impulse ≥ 100 ms displayed excellent reliability (ICC = 0.91–1.00; CV = 2.3–11.6%), however whilst RTD and impulse ≤ 50 ms displayed moderate-to-good ICC reliability (ICC = 0.57–0.77), CV’s were large (39.8–46.6%). Therefore, due to the large variation within- and between-sessions, epochs ≤ 50 ms were not statistically analysed in the present study.

### Sample size

Effect sizes (Cohen’s *d*) were calculated from previous studies employing similar interventions from mean changes in strength or muscle soreness (Macintyre et al. 1996; Kubo et al. 2005) to determine the necessary sample size for statistical power and to ensure adequate statistical power for all analyses. A priori power analysis using G*Power (v.3.1 Düsseldorf, Germany) was conducted using strength (i.e. the variable with the smallest effect size) using the following parameters; α = 0.05, β = 0.20, *d* = 1.59, attrition = 20%. The analysis revealed a minimum sample size of eight participants, with 13 participants recruited for the study to account for potential participant attrition and data loss.

## Results

### Eccentric exercise sessional data

There were no significant differences in the average force production (560 ± 65 vs. 605 ± 72 N) or mechanical work performed (11.62 ± 1.62 vs. 12.23 ± 1.66 kJ) between the eccentric exercise sessions in Bout 1 and Bout 2, indicating a similar exercise intensity and volume across both sessions.

### Neuromuscular function

The two-way repeated measures ANOVA revealed no interaction effect between time and bout for metrics of balance (*F* = 0.28–1.86, *P* = 0.13–0.89, η_p_^2^ = 0.02–0.13), and no main effects of time (*F* = 0.10–2.41, *P* = 0.06–0.98, η_p_^2^ = 0.01–0.17) or bout (*F* = 0.19–3.88, *P* = 0.07–0.66, η_p_^2^ = 0.02–0.24) (Table 1). Similarly, no interaction effect between time and bout was revealed for maximal isometric torque (*F* = 0.12, *P* = 0.97, η_p_^2^ = 0.01), and no main effects of time (*F* = 0.12, *P* = 0.07, η_p_^2^ = 0.01) or bout (*F* = 1.95, *P* = 0.19, η_p_^2^ = 0.14) (Fig. 4a).

**Table 1:** Metrics of balance normalised to pre-exercise (100%) in the respective bout (mean ± SE)

	Bout 1				Bout 2			
Time (hr)	0	24	48	72	0	24	48	72
Measurement (%)								
EO AP sway	117 ± 16	125 ± 22	110 ± 23	127 ± 18	104 ± 6	109 ± 7	101 ± 9	110 ± 6
EO ML sway	104 ± 7	104 ± 10	102 ± 13	109 ± 6	109 ± 9	111 ± 7	113 ± 11	124 ± 10
EO velocity	98 ± 7	93 ± 6	92 ± 5	93 ± 6	98 ± 4	103 ± 3	102 ± 4	102 ± 3
EO 95% ellipse	112 ± 15	108 ± 12	99 ± 12	120 ± 18	109 ± 12	113 ± 12	119 ± 13	118 ± 6
EC AP sway	98 ± 7	115 ± 13	102 ± 4	106 ± 13	99 ± 5	103 ± 5	103 ± 9	106 ± 6
EC ML sway	101 ± 8	109 ± 6	109 ± 7	98 ± 6	99 ± 10	105 ± 7	105 ± 15	105 ± 10
EC velocity	97 ± 4	101 ± 4	98 ± 3	100 ± 5	98 ± 3	98 ± 3	99 ± 5	99 ± 4
EC 95% ellipse	99 ± 7	120 ± 16	111 ± 6	111 ± 16	88 ± 8	100 ± 7	116 ± 14	104 ± 11
Max. AL	105 ± 10	109 ± 6	116 ± 8	109 ± 8	101 ± 9	93 ± 4	94 ± 5	101 ± 5

*SE* standard error, *hr* hours, *EO* eyes open, *EC* eyes closed, *AP* anteroposterior, *ML* mediolateral, *Max. AL* maximum anterior lean

**Fig. 3:**
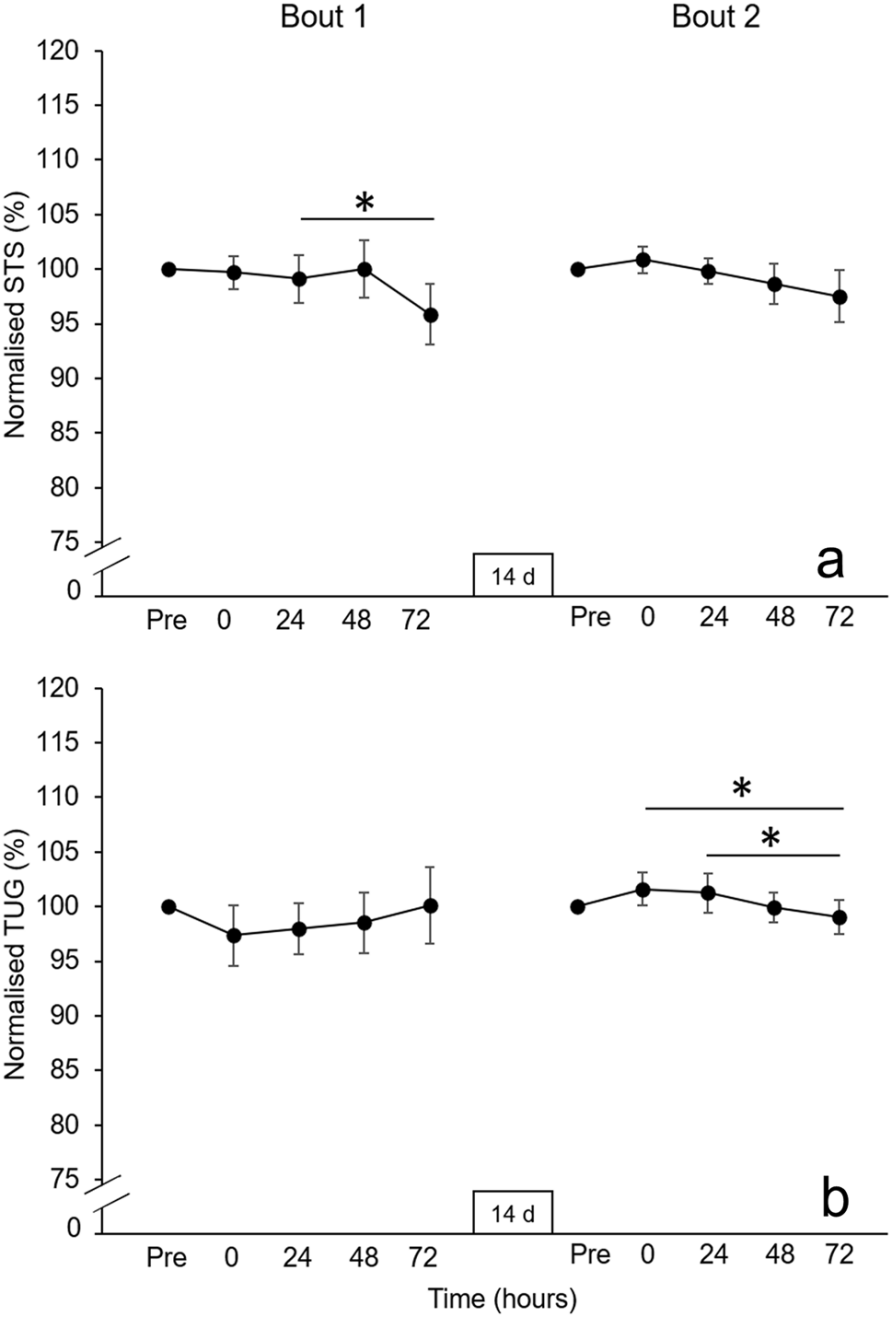
Panel **a** displays STS time normalised to pre-exercise (100%) in the respective bout and panel **b** displays TUG normalised to pre-exercise (100%) in the respective bout (mean ± SE). *P ≤ 0.05. STS performance was significantly faster at 72 hr post-exercise compared to 24 hr post-exercise in Bout 1. TUG performance was significantly faster at 72 hr post-exercise compared to immediately post- and 24 hr post-exercise in Bout 2

No interaction effect between time and bout was revealed for STS (*F* = 0.72, *P* = 0.48, η_p_^2^ = 0.06), however main effects of time (*F* = 3.51, *P* = 0.01, η_p_^2^ = 0.23) and bout (*F* = 12.07, *P* = 0.01, η_p_^2^ = 0.50) were detected (Fig. 3a). In Bout 1, participants were significantly faster whilst performing the STS at 72 hr post-exercise compared to 24 hr post-exercise (24.1 ± 4.94 vs. 23.2 ± 4.30 s, -3.7 ± 4.9% [-0.90 ± 1.26 s]; *P* = 0.03, *d* = 0.71), whereas no differences were found in Bout 2 (*P* > 0.05).

The Friedman test revealed no main effect of time for TUG in Bout 1 (χ^2[4]^ = 5.97, *P* = 0.20) but did reveal a main effect of time in Bout 2 (χ^2[4]^ = 10.58, *P* = 0.03). In Bout 2, TUG was significantly faster 72 hr post-exercise (7.00 ± 0.31 s) than immediately post-exercise (7.16 ± 0.30 s; − 2.5 ± 3.3% [-0.16 ± 0.22 s]; *P* = 0.01*, r* = 0.74) and 24 hr post-exercise (7.12 ± 1.03 s, − 2.0 ± 6.6% [-0.12 ± 0.45 s]; *P* = 0.01, *r* = 0.76) (Fig. 3b).

No interaction effects (*F* = 0.01–1.27, *P* = 0.30–0.95, η_p_^2^ = 0.07–0.10) or main effects of time (*F* = 0.30–2.01, *P* = 0.101–0.88, η_p_^2^ = 0.02–0.14) or bout (*F* = 0.03–2.37, *P* = 0.15–0.88, η_p_^2^ = 0.00–0.17) were revealed for the majority of RTD metrics, except for a main effect of time for RTD_0-200_ (*F* = 2.63, *P* = 0.05, η_p_^2^ = 0.18). In Bout 1, RTD_0-200_ was significantly greater at 72 hr post-exercise than immediately post-exercise, 24 hr post-exercise, and 48 hr post-exercise (Table 2). RTD_0-200_ was significantly lower in Bout 2 at 24 hr post-exercise compared to pre-exercise and immediately post-exercise.

**Table 2:** Metrics of explosive ability normalised to pre-exercise (100%) in the respective bout (mean ± SE)

	Bout 1				Bout 2			
Time (hr)	0	24	48	72	0	24	48	72
Measurement (%)								
RTD_0-100_	97 ± 5	106 ± 12	107 ± 11	117 ± 12	104 ± 8	88 ± 8	108 ± 15	108 ± 15
RTD_0-150_	99 ± 6	104 ± 9	107 ± 10	114 ± 10	101 ± 4	88 ± 6	101 ± 9	100 ± 8
RTD_0-200_	99 ± 5	102 ± 7	104 ± 8	111 ± 8^b,c,d^	100 ± 4	91 ± 4^a,b^	101 ± 7	100 ± 6
RTD_0-250_	99 ± 5	101 ± 6	104 ± 7	110 ± 7	101 ± 4	94 ± 3	102 ± 7	103 ± 6
RTD_0-300_	98 ± 4	101 ± 5	103 ± 5	108 ± 5	100 ± 3	98 ± 3	106 ± 5	103 ± 5
RTD_100-200_	101 ± 7	101 ± 9	105 ± 8	110 ± 9	99 ± 5	98 ± 5	103 ± 5	95 ± 5
RTD_Peak_	96 ± 4	98 ± 5	100 ± 5	106 ± 5	103 ± 4	95 ± 3	103 ± 6	101 ± 6
Impulse_0-100_	97 ± 5	107 ± 12	107 ± 10	119 ± 11	103 ± 9	87 ± 16	108 ± 16	109 ± 15
Impulse_0-150_	99 ± 6	104 ± 9	107 ± 10	107 ± 9	100 ± 5	88 ± 6	101 ± 10	100 ± 8
Impulse_0-200_	99 ± 5	102 ± 7	104 ± 8	111 ± 8	100 ± 4	91 ± 4	101 ± 8	101 ± 6
Impulse_0-250_	99 ± 5	102 ± 6	104 ± 7	110 ± 6^b,c^	100 ± 4	92 ± 4^b,d,e^	102 ± 17	103 ± 6
Impulse_0-300_	98 ± 4	102 ± 5	103 ± 5	109 ± 5	99 ± 3	89 ± 7^a^	104 ± 6	104 ± 5

*SE* standard error, *hr* hours, *RTD* rate of torque development

^a^Significant difference to pre-exercise

^b^Significant difference to immediately post-exercise

^c^Significant difference to 24 hr post-exercise

^d^Significant difference to 48 hr post-exercise

^e^Significant difference to 72 hr post-exercise in the respective bout; *P* < 0.05

No interaction effects (*F* = 0.71–1.34, *P* = 0.59–0.32, η_p_^2^ = 0.06–0.10) or main effects of time (*F* = 1.89–2.51, *P* = 0.06–0.13, η_p_^2^ = 0.14–0.17) or bout (*F* = 0.01–1.70, *P* = 0.22–0.91, η_p_^2^ = 0.00–0.12) were revealed for the majority of metrics of impulse, except for Impulse_0-250_ (*F* = 2.82, *P* = 0.04, η_p_^2^ = 0.19) and Impulse_0-300_ (*F* = 2.90, *P* = 0.03, η_p_^2^ = 0.19) over time. In Bout 1, Impulse_0-250_ was significantly greater at 72 hr post-exercise compared to immediately post-exercise and 24 hr post-exercise (Table 2). In bout 2, Impulse_0-250_ was significantly lower at 24 hr post-exercise compared to immediately post-exercise, 48 hr, and 72 hr post-exercise. There were no significant differences for Impulse_0-300_ between time points in Bout 1, however in Bout 2 Impulse_0-300_ was significantly reduced at 24 hr post-exercise compared to pre-exercise levels.

**Fig. 4:**
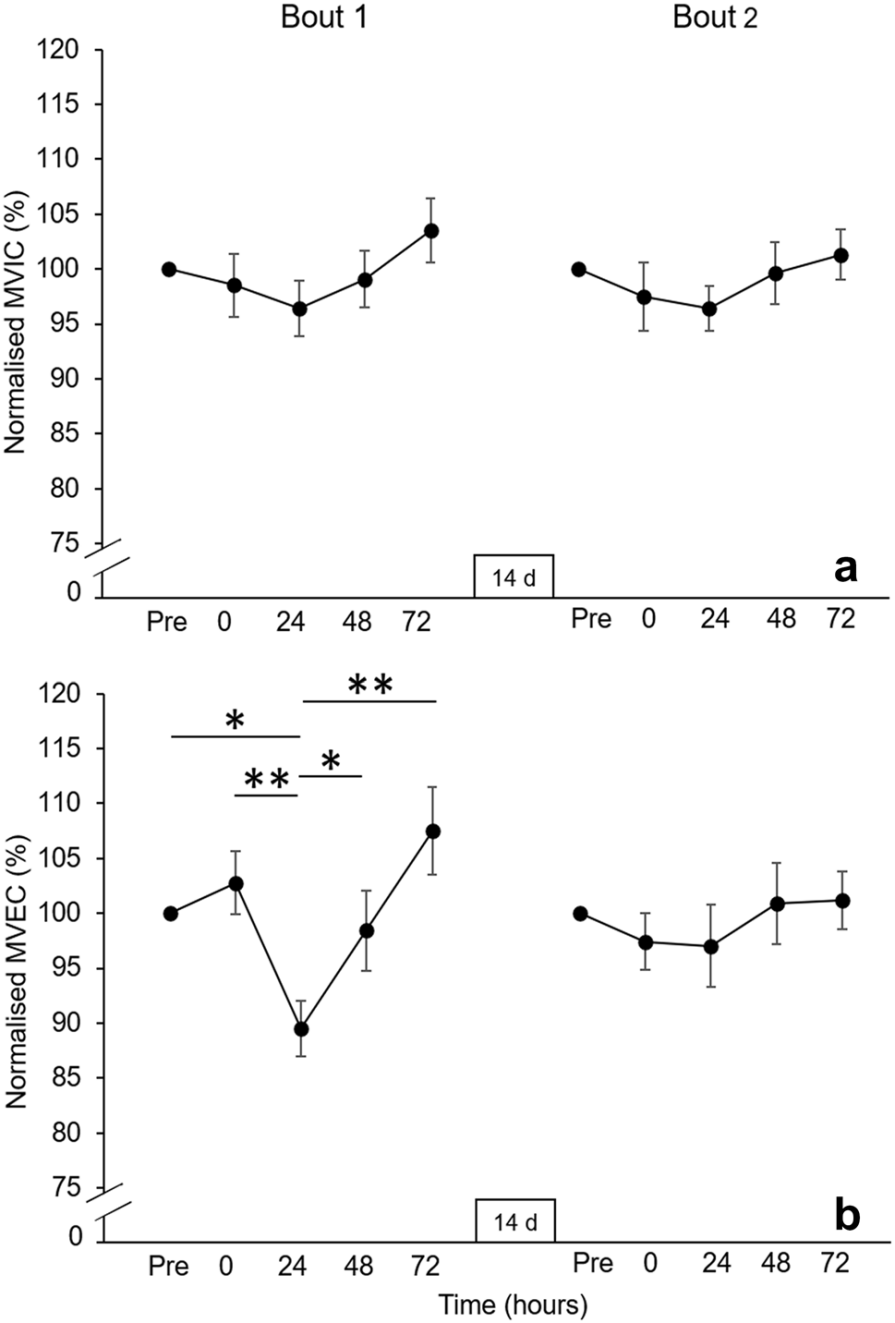
Panel a displays maximal isometric torque normalised to pre-exercise (100%) in the respective bout and panel b displays maximal eccentric force normalised to pre-exercise (100%) in the respective bout (mean ± SE). *P ≤ 0.05, **P ≤ 0.001. Maximal eccentric force was significantly reduced at 24 hr post-exercise in Bout 1 compared to all other timepoints within the respective bout, but no changes were evident in Bout 2

An interaction effect was revealed for maximal eccentric force (*F* = 2.69, *P* = 0.04, η_p_^2^ = 0.18), alongside main effects of time (*F* = 7.45, *P* < 0.001, η_p_^2^ = 0.38) and bout (*F* = 6.10, *P* = 0.03, η_p_^2^ = 0.34) (Fig. 4b). Maximal eccentric force at 24 hr post-exercise (1065 ± 330 N) was significantly lower in Bout 1 compared to pre-exercise (1218 ± 457 N, − 12.6 ± 9.2% [− 153 ± 160 N]; *P* = 0.01, *d* = 0.96), immediately post-exercise (1230 ± 410 N, − 13.4 ± 7.6% [− 165 ± 137 N]; *P* < 0.001, *d* = 1.20), 48 hr (1172 ± 371 N, − 9.2 ± 13.9% [− 107 ± 158 N]; *P* = 0.03, *d* = 0.68) and 72 hr post-exercise (1278 ± 399 N, − 16.6 ± 8.9% [− 212 ± 144 N]; *P* < 0.001, *d* = 1.48). The index of protection for maximal eccentric force calculated between bouts from pre-exercise to 24 hr post-exercise was 76.8%.

### Perceptual measures

The Friedman test revealed a main effect of time for palpation muscle soreness in Bout 1 (χ^2[4]^ = 15.64, *P* = 0.04) but not Bout 2 (χ^2[4]^ = 5.35, *P* = 0.25). Palpation muscle soreness significantly increased from pre-exercise to 24 hr post-exercise in Bout 1 (0.23 ± 0.13 vs. 0.90 ± 0.29; *P* = 0.02, *r* = 0.78). No main effect of time was revealed for movement muscle soreness in either bout (χ^2[4]^ = 5.67–7.90, *P* = 0.10–0.23).

A main effect of time was revealed in Bout 1 for RPE whilst performing the STS test (χ^2[4]^ = 23.39, *P* < 0.001) but not Bout 2 (χ^2[4]^ = 8.56, *P* = 0.07). Within Bout 1, RPE during the STS test significantly increased from pre-exercise (0.90 ± 0.27) to immediately post-exercise (1.70 ± 0.34; *P* < 0.001, *r* = 1.40), 24 hr (1.60 ± 0.30; *P* < 0.001, *r* = 1.21), 48 hr (1.60 ± 0.31; *P* = 0.00, *r* = 1.22), and 72 hr (1.44 ± 0.36; *P* = 0.01, *r* = 1.09) post-exercise. A main effect of time for RPE whilst performing the TUG test was revealed in Bout 1 (χ^2[4]^ = 13.36, *P* = 0.01) and Bout 2 (χ^2[4]^ = 31.42, *P* < 0.001). Within Bout 1, RPE during the TUG test significantly increased from pre-exercise (0.08 ± 0.08) to immediately post-exercise (0.67 ± 0.22; *P* = 0.03, *r* = 0.37) and 72 hr (0.67 ± 0.21; *P* = 0.02, *r* = 0.42) post-exercise. Within Bout 2, RPE during the TUG test significantly increased from pre-exercise (0.38 ± 0.21) to 0 hr (0.78 ± 0.27; *P* < 0.001, *r* = 0.69), 24 hr (0.49 ± 0.18; *P* = 0.02, *r* = 0.42), 48 hr (0.44 ± 0.18; *P* < 0.001, *r* = 0.63), and 72 hr (0.33 ± 0.62; *P* < 0.001, *r* = 0.61) post-exercise. No significant difference was revealed between RPE whilst performing the eccentric exercise (*Z* = 18.50, *P* = 0.19).

## Discussion

Muscular strength is a reliable indirect marker of EIMD (Warren et al. 1999; Hyldahl and Hubal 2014) associated with functional ability (Skelton et al. 1994; Bouchard et al. 2011) and was a key outcome measure in the present study. Despite no change in isometric strength after either Bout 1 or 2 at any time point, reductions in eccentric strength (− 13%) were evident at 24 hr post-exercise in Bout 1, with no significant change evident after Bout 2. Eccentric muscle actions absorb mechanical work to decelerate a body in motion (Lindstedt et al. 2001), with the lower-limb muscles eccentrically contracting to control the centre of mass during a slip, trip, or fall. In the present study, eccentric strength was assessed via an alternating unilateral multi-joint movement (eccentric single leg press) that measured the combined strength of the lower-limb extensor muscle groups (hip extensors, knee extensors, and plantar flexors). To react to a trip, the base of support must be rapidly increased, which is achieved by taking an anterior step (Karamanidis et al. 2020) and using the lower-limb extensor muscles to stabilise the centre of mass. Due to the age-related reduction in lower-limb strength, the ability to rapidly increase the base of support and stabilise the centre of mass is hindered in older adults compared to their younger counterparts (Suptitz et al. 2013), so the decrease in eccentric strength 24 hr post-exercise may exacerbate this ability and further increase the risk of falling. However, these findings indicate that low-volume and low-intensity eccentric exercise results in a minimal impairment of strength, which is rapidly resolved after an initial bout and is absent in a subsequent bout. Nonetheless, clinicians could advise patients to proceed with caution during ambulation in the 24 hr after an initial exposure to this exercise.

Postural instability is associated with falls (Overstall et al. 1971; Johansson et al. 2017), even in physically active community-dwelling older adults (Zhou et al. 2017). The capacity to maintain an upright stance is largely associated with proprioception (Lee et al. 2006; Wang et al. 2016) and is therefore, dependent upon the ability of the central nervous system to respond appropriately to afferent motor signals (Nashner 1976). Reductions in proprioceptive ability will compromise balance and are associated with the magnitude of strength-loss induced by fatigue (Proske 2019). Given that isometric strength was not compromised in the present study, disruption to proprioceptive capacity is unlikely and may explain the lack of change in any balance metrics in the days following an acute bout of eccentric exercise. The lack of change in any balance metric may be indicative that the eccentric exercise did not induce muscle fatigue, possibly a consequence of the low metabolic demand (Hoppeler 2016). To the authors’ knowledge, only Hill et al. (2020) have assessed the short-term effects of eccentric exercise on older adults’ postural stability, reporting that immediately after 30 min of downhill walking their postural stability was unaffected, which concurs with the findings of the present study. Conversely, Hill et al. (2020) also reported that balance was impaired at 24 and 48 hr post-exercise, although these disparate findings are likely a consequence of the exercise volume and/or type of eccentric exercise as Hill et al. (2020) prescribed a substantial volume to induce muscle damage (downhill walking for 30 min), unlike the present study that utilised 5 min on a recumbent stepper. Collectively, these data indicate that the volume of exercise may be an important variable to control when prescribing specific eccentric exercise modes for older adults and that multi-joint low-volume and submaximal eccentric exercise can be performed by older adults without short-term reductions in static balance.

While muscle strength and balance are commonly examined in age-related research, a fall often occurs in ≤ 120 ms (Lockhart 2013) and therefore, rapid force generating capacity and power may be more appropriate measures to determine the potential short-term negative effects of an intervention on fall risk. Most of the explosive measures (RTD_0-100_, _0–150_, _0–250_, _0–300_, _100–200_, _Peak_, Impulse_0-100_, _0–150_, _0–200_) and functional ability (STS and TUG tests) were not compromised, providing further support that the eccentric exercise elicited minimal disruption to contractile ability. While RTD_0-200_, Impulse_0-250_, and Impulse_0-300_ were reduced at 24 hr post-exercise in Bout 2, these are considered late-phase explosive measures that may have been affected because eccentric exercise predominantly recruits (and therefore damages) fast-twitch muscle fibres (Friden et al. 1983) commonly associated with late-phase explosive capacity. However, RTD is a complex and multi-faceted parameter (Maffiuletti et al. 2016) and because the mechanisms responsible were beyond the scope of this study, it is unknown why these alterations occurred. Nonetheless, as a fall often occurs in ≤ 120 ms (Lockhart 2013), the reductions evident only in late phase RTD likely have little influence on the risk of falling in older adults, providing further support for eccentric exercise being a suitable exercise modality to prescribe to older adults who are often at a higher risk of falling.

Whilst performing the STS and TUG tests, RPE was increased after Bout 1 for 72 hr post-exercise. Muscle soreness was also elevated following Bout 1, which may have influenced perceptions of exertion as soreness was aligned with RPE during the STS test for up to 72 hr post-exercise in Bout 1. The low-intensity and low-volume exercise completed in the study likely limited the elevation in pain post-exercise as both perceptual increases were only by one point on their respective scales, so it is questionable as to whether these will have an influence on habitual activity levels. However, two studies (Lavender and Nosaka 2006, 2008b) have found that older participants generally report lower levels of soreness compared to their younger counterparts, which may be attributable to age-related alterations in nociception (Gibson and Farrell 2004) and therefore, age may be a confounding variable for the minimal soreness reported. Similarly, lower-limb muscle groups are less prone to muscle soreness (Chen et al. 2011; Hyldahl and Hubal 2014), thus the muscle groups assessed may also explain the limited soreness reported. Regardless, the eccentric exercise prescribed had minimal effect on perceptual measures of soreness and exertion, which may be inter-related but could influence attitudes towards physical activity. Furthermore, RPE did not significantly increase following Bout 2 indicative of a RBE that has been evidenced to influence perceptual measures (Burt et al. 2013; Hyldahl et al. 2017) even after low-intensity eccentric work (Chen et al. 2013). It is possible that the RBE positively affects perceptual measures of discomfort and/or effort and therefore, to improve adherence to exercise programmes, practitioners could advise patients that discomfort following the initial exposure may be alleviated in subsequent sessions. However, this protective effect from low-intensity exercise may only last two weeks (Chen et al. 2012a), therefore practitioners/clinicians should commence an eccentric training programme within this time frame following the initial exposure to maximise the safety and comfort of patients.

There are several limitations of the present study. Firstly, given that the eccentric exercise was performed unilaterally on a stepper ergometer, it was essential that participants were familiarised and performed a practice session on the machine to enable correct technique and to ensure safety. Whilst the practice session was performed briefly at a very low perceived intensity (~ 25%), it may have preconditioned the participants to the eccentric exercise (Chen et al. 2012b; Maeo et al. 2017) and partially elicited the RBE before Bout 1. Regardless, safety and proper technique are both essential and cannot be avoided, thus it is likely that future works involving novel training devices will need to consider this during study conception. Secondly, the sample in the present study included homogenous, active, and functionally capable individuals and the magnitude of EIMD may be greater in clinical populations with comorbidities such as individuals suffering from frailty who are also at a greater risk of falling (Fried et al. 2001). However, the sample also consisted of both sexes to improve the generalisability of the findings, thus future research could examine whether sex influences EIMD and the RBE following multi-joint eccentric exercise in older adults. Thirdly, poor reliability of the very early-phase explosive capacity (≤ 50 ms) data prevented our ability to confidently report findings of these epochs. As these epochs may be more closely associated with the ability to counteract a slip, trip, or fall, further research is warranted using specialised equipment that provide more reliable data (e.g. custom-built rigid dynamometers) to fully elucidate the effects of multi-joint eccentric exercise on fall-risk factors in older adults. Finally, as maximal eccentric force was the only metric that was significantly compromised during Bout 1, which was tested on the same machine (Eccentron) used to perform the eccentric exercise, the reduction may potentially be due to a test-specific transfer effect. Therefore, future research should consider utilising alternative methods of assessment that are different to the exercise where possible (e.g. uni-joint eccentric torque as a measure of eccentric strength) to eliminate the potential for test-specific transfer effects. Therefore, conclusions may only be generalisable to community-dwelling older adults with further research warranted to determine (i) the short-term effects and appropriateness of prescribing eccentric exercise to more physically compromised populations and (ii) the acute effects of multi-joint eccentric exercise on very early-phase RTD and uni-joint eccentric torque.

In conclusion, submaximal multi-joint eccentric exercise elicits negligible changes in neuromuscular function when prescribed at a low intensity and volume (50% of maximal eccentric force for 5 min). While eccentric strength decreased at 24 hr post-exercise in Bout 1, the RBE eliminated this reduction following Bout 2, providing further evidence that the RBE can allow for progression into an eccentric training programme. These data provide further support that this type of resistance training is suitable to prescribe to functionally capable older individuals to combat age-related neuromuscular degeneration. Further research is warranted to determine the short-term suitability of this exercise modality in physically compromised populations, particularly those with neuromusculoskeletal comorbidities. Finally, additional investigations into to the progression of workload following the initial bout of exercise are required to provide more evidence around the RBE to inform the design of progressive eccentric exercise interventions in clinical populations.

## Data Availability

The datasets that support the findings of this study are openly available PURE at http://doi.org/10.24339/3cf619ad-ca74-4a3e-91f1-8d52a8cc6d04, reference number 42303102.

## Abbreviations

ANOVA: Analysis of variance
CV: Coefficient of variation
*d*: Cohen’s *d*
DOMS: Delayed onset muscle soreness
EIMD: Exercise-induced muscle damage
ICC: Intraclass correlation coefficient
RBE: Repeated bout effect
RPE: Rate of perceived exertion
RTD: Rate of torque development
SD: Standard deviation
SE: Standard error
STS: Sit-to-stand
TUG: Timed up-and-go
